# Designing High Performance Carbon/ZnSn(OH)_6_-Based Humidity Sensors

**DOI:** 10.3390/s24113532

**Published:** 2024-05-30

**Authors:** Min Zhang, Hongguang Jia, Shuying Wang, Zhenya Zhang

**Affiliations:** School of Physics Science and Technology, Xinjiang University, Urumqi 830046, China

**Keywords:** carbon/ZnSn(OH)_6_, humidity sensor, surface hydroxyl group

## Abstract

In this work, pure phase and carbon/ZnSn(OH)_6_ samples were synthesized by a hydrothermal method. The composite sample’s structure, morphology, and functional groups were investigated by X-ray diffraction, field-emission scanning electron microscopy, transmission electron microscopy, and Fourier transform infrared spectroscopy. Subsequently, ZnSn(OH)_6_ samples were modified with different carbon contents, and their humidity-sensing properties were investigated. The introduction of carbon increased the specific surface area of pure ZnSn(OH)_6_ samples, thus significantly improving the sensors’ humidity sensing response. The C10-ZnSn(OH)_6_ sensor exhibited a high response, up to three orders of magnitude, a humidity hysteresisof 13.5%, a fast response time of 3.2 s, and a recovery time of 24.4 s. The humidity sensor’s possible humidity sensing mechanism was also analyzed using the AC complex impedance puissance method with a simulated equivalent circuit. These results revealed that ZnSn(OH)_6_ can effectively detect ambient humidity and that the introduction of carbon significantly improves its humidity-sensing performance. The study provides an effective strategy for understanding and designing ZnSn(OH)_6_-based humidity sensors.

## 1. Introduction

Sensors have attracted great interest in many fields due to their unique, excellent performance and broad application prospects. Humidity sensors are widely used in respiratory monitoring, industrial corrosion, agriculture, environmental analysis, and other fields [1,2,3,4]. As the core of humidity sensors, the research has been focused on developing sensitive substrates, including metal oxides, polymers, natural nanoclay, cellulose, metal hydroxides, and some two-dimensional materials (black phosphorus, black arsenic), etc. [5,6,7,8,9,10,11].

ZnSn(OH)_6_ is a metal hydroxide in which metal atoms are octahedrally coordinated with oxygen atoms to form a Sn(OH)_6_ polyhedra and a Zn(OH)_6_polyhedra. These polyhedra share an O-angle connection to create a structural framework, forming a face-centered cubic crystal structure (FCC). ZnSn(OH)_6_ is non-toxic, easy to prepare, andlow-cost; therefore, it is widely applied in photocatalysis [12], gas sensors [13], flame retardant, smoke suppression [14], negative materials for ion batteries [15], inorganic fillers [16], and many other fields. Humidity detection is closely linked to the material’s microstructure, crystal defects, specific surface area, active sites, and surface hydrophilic groups [17,18]. Our previous research reported that the main lattice of ZnSn(OH)_6_ contains abundant hydroxyl groups essential for the adsorption and desorption of water molecules [9]. The sensing mechanism of humidity sensors involves the water molecules’ adsorption by hydrophilic functional groups on their surface, causing an increase in ionic conductivity and a change in resistive properties (resistance or capacitance). Thus, a ZnSn(OH)_6_ crystal would respond highly to humidity signals [19]. Additionally, its wider band gap (4.35 eV) [20] allows for higher stability in signal detection, broadening its application prospects in microelectronics.

The materials’ sensing performance is closely related to the adsorption and desorption of chemical signals, as well as the catalytic activity on the sensing material surface. Modification with precious metals, such as Pt, Ag, or Au, onto sensitive substrate is still a meaningful way to improve the chemical activity [21,22,23]. However, using precious metal modification increases the preparation cost and causes metals to aggregate during loading, having adverse effects on sensing performance improvement. Therefore, developing other substitutes for precious metals has been a focus. Carbon-based materials, such as carbon nanotubes, graphene, and other carbon-based materials, have received significant attention, and exhibit unique physical structures and excellent electrical properties [8,24]. Carbon-based materials have been the most promising alternative to noble metal modification [25]. In this research, we employed a simple and effective wet chemical method for synthesizing a carbon/ZnSn(OH)_6_ octahedron. The fabricated carbon/ZnSn(OH)_6_ humidity sensor demonstrates excellent humidity detection performance. The possible humidity sensing mechanism of sensors is analyzed using the AC complex impedance spectrum with simulated equivalent circuits. The study results provide evidence to explore and design ZnSn(OH)_6_-based humidity-sensitive materials.

## 2. Experimental Details

### 2.1. Materials

Zinc acetate (Zn(AC)_2_·2H_2_O), tin tetrachloride pentahydrate (SnCl_4_·5H_2_O), sodium hydroxide (NaOH), dextran (C6nH10nO5n), ethanol (C_2_H_5_OH), lithium chloride (LiCl), magnesium chloride (MgCl_2_), potassium carbonate (K_2_CO_3_), magnesium nitrate (Mg(NO_3_)_2_), copper chloride (CuCl_2_), sodium chloride (NaCl), potassium chloride (KCl), and potassium nitrate (KNO_3_) were purchased from Sinopharm Chemical Reagent Co., Ltd., Beijing, China. All of the reagents used in this experiment were of analytical grade.

### 2.2. Synthesis of Carbon/ZnSn(OH)_6_

**Figure 1:**
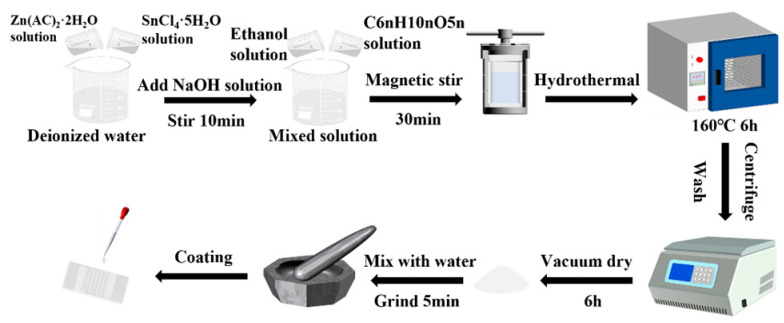
Preparation process diagram of carbon/ZnSn(OH)_6_ sensor.

Firstly, 0.7 g SnCl_4_·5H_2_O and 0.44 g Zn(AC)_2_·2H_2_O were dissolved in 5 mL deionized water under continuous stirring. After 10 min, 8 mL NaOH solution (3 M) was slowly added dropwise to the above solution, and then 10 mL 40% aqueous ethanol solution was added. Subsequently, 5 mL C6nH10nO5n solution was dropped into the above mixture and stirred for 30 min. The mixed solution was transferred to a 50mL high-pressure reactor and heated to 160 °C for 6 h. After the reaction, the residuals were cooled naturally, and washed with ethanol and deionized water several times. Finally, the precipitate was dried under vacuum at 60 °C for 6 h. The final product obtained was ground to fine powder. The mass ratios of C6nH10nO5n to ZnSn(OH)_6_ were 0 wt%, 5 wt%, 10 wt%, and 15 wt%, and labelled as ZnSn(OH)_6_, C5-ZnSn(OH)_6_, C10-ZnSn(OH)_6_, and C15-ZnSn(OH)_6_, respectively (Figure 1).

### 2.3. Materials Characterization

The samples’ crystal structure and composition were determined by X-ray diffractometer (XRD, D8 advance, Bruker, Karlsruhe, Germany) with a Cu Kα radiation (λ = 1.5418 nm) in the 2θ range of 10–80°. The micro-morphology of samples was observed by a field emission scanning electron microscope (SEM, Simgma 300, ZEISS, Oberkochen, Germany) at an accelerating voltage of 5 kV. The internal nanostructures and elemental distribution of samples were characterized by transmission electron microscopy (TEM, TecnaiG2 F20, FEI, Hillsborough, OR, USA) and energy spectrometry (EDS, JEOL JEM-3010, Tokyo, Japan) at an operating voltage of 200 kV.

The elemental compositions of materials were analyzed by X-ray photoelectron spectroscopy (XPS, Thermo ESCALAB 250Xi, Waltham, MA, USA) using monochromatic Al Kα radiation as an excitation source. Fourier transform infrared spectroscopy (FT-IR) spectra of samples were recorded with a Nicolet IS5 spectrometer in the 400–4000 cm^−1^ range and averaged 32 times. A KBr pellet was used for FT-IR measurements. The N_2_ adsorption/desorption experiments were performed on an automatic gas adsorption device (ASAP2460, Mack, Atlanta, GA, USA) at 77 K. The surface areas were calculated using the Brunauer–Emmett–Teller (BET) equation. The humidity response was analyzed by an electrochemical workstation (ZAHNER-IM6EX, Kronach, Germany).

### 2.4. Fabrication and Measurement of Humidity Sensors

Firstly, the prepared ZnSn(OH)_6_, C5-ZnSn(OH)_6_, C10-ZnSn(OH)_6_, and C15-ZnSn(OH)_6_ samples were placed in a mortar, and an appropriate amount of deionized water was added, and ground for 5 min. Then, the mixture was coated on an Ag-Pd interdigital electrode and dried naturally at room temperature overnight.

Subsequently, saturated salt solutions of LiCl, MgCl_2_, K_2_CO_3_, Mg(NO_3_)_2_, CuCl_2_, NaCl, KCl, and KNO_3_ were prepared in chambers to obtain humidity environments of 11%, 33%, 43%, 54%, 64%, 75%, 85%, and 95% RH, respectively. The electrical performance of sensors was measured using an electrochemical workstation (ZAHNER-IM6EX, Kronach, Germany). The test voltage was set to 1 V AC at 40–100 kHz. The devices were placed into the chamber sequentially (11–95% RH) to obtain the relationship between the response of the sensor and relative humidity. A commercial electronic thermohygrometer (FY-12, Vanke Sheng, Shenzhen, China, 1–99% RH) was used to monitor relative humidity environment in the chamber. These experiments were conducted at room temperature at 30% RH at standard atmospheric pressure.

## 3. Results and Discussion

### 3.1. Structure and Morphology Analysis

**Figure 2:**
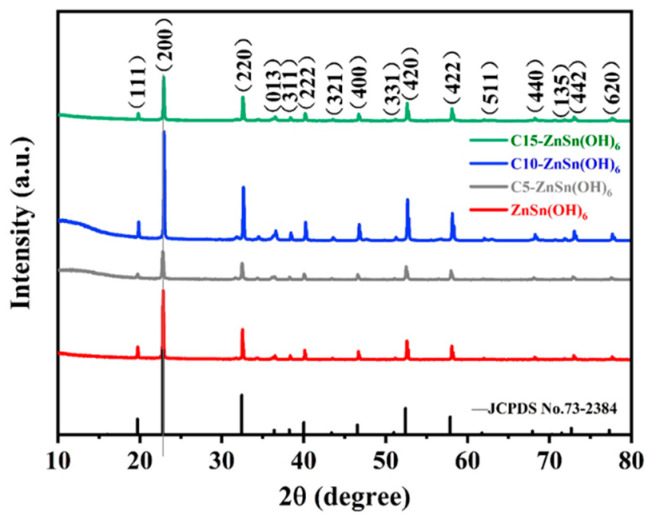
The XRD patterns of ZnSn(OH)_6_, C5-ZnSn(OH)_6_, C10-ZnSn(OH)_6_, and C15-ZnSn(OH)_6_ samples.

The crystal structures of pure phase and carbon/ZnSn(OH)_6_ samples were determined by XRD analysis (Figure 2). By spectral comparison, the standard card corresponding to ZnSn(OH)_6_ is identified as JCPDS.No73-2384. The intensity of diffraction peaks is strong, indicating that the synthesized ZnSn(OH)_6_ exhibits high crystallinity. No diffraction peaks corresponding to the carbon are observed for the carbon/ZnSn(OH)_6_ samples, indicating that carbon modification did not change the original crystal structure of ZnSn(OH)_6_. The full peaks of carbon-modified samples do not shift towards high or low angles, revealing that their lattice spacing stays consistent with the pure one. The structure of crystal materials directly determines their electrical, optical, and other properties, which in turn affect the sensitivity, stability, and other performance of sensors. As shown in Figure 2, the crystallinity of C10-ZnSn(OH)_6_ samples is better than that of C5-ZnSn(OH)_6_ and C15-ZnSn(OH)_6_ samples, which may directly affect the response of their humidity sensors.

**Figure 3:**
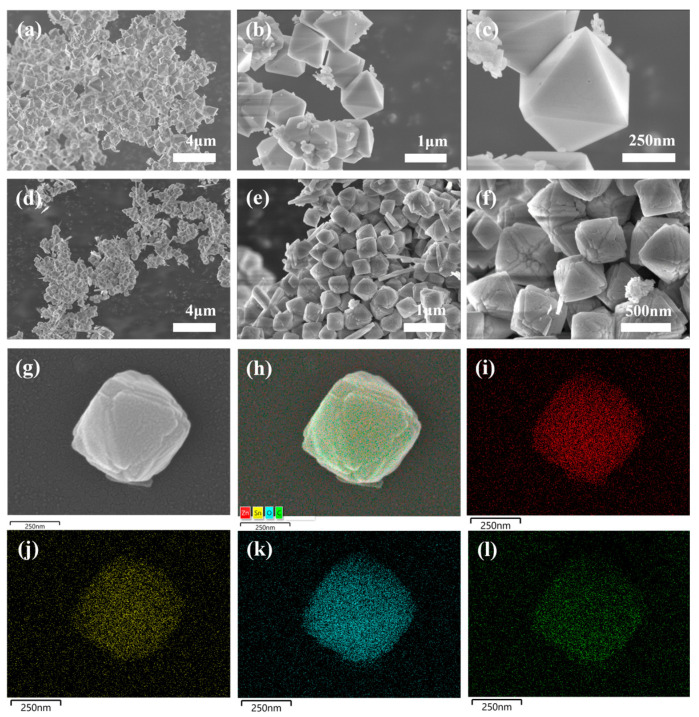
(**a**–**c**) SEM images of pure ZnSn(OH)_6_ samples at different magnifications; (**d**–**g**) SEM images of C10-ZnSn(OH)_6_ samples at different magnifications; and (**h**–**l**) mapping images of all elements, Zn, Sn, O, and C elements in C10-ZnSn(OH)_6_ samples, respectively.

Samples’ surface morphology and microstructure are essential in their humidity sensing performance. Figure 3 illustrates the morphology of ZnSn(OH)_6_ and C10-ZnSn(OH)_6_ samples at high and low magnification and the mapping images of C10-ZnSn(OH)_6_ samples. ZnSn(OH)_6_ morphology is observed as an octahedron shape with a dimension around 1 μm (Figure 3a–c). SEM images of ZnSn(OH)_6_ after carbon inducing is shown in Figure 3d–g. Each face of the octahedron is approximately a regular triangle. The morphology of the C10-ZnSn(OH)_6_ sample is similar to that of pure ZnSn(OH)_6_, and the octahedron-like morphological structure remains almost unchanged. Moreover, the C10-ZnSn(OH)_6_ crystal grows, reducing the aggregation of grains. Obvious cracks were observed on the surface of the C10-ZnSn(OH)_6_; the relatively rough surface of it would result in a large surface area.

Zn, Sn, O, and C elements in C10-ZnSn(OH)_6_ are detected (Figure 3h–l is the mapping image of all elements), and the elements are evenly distributed; in particular, Figure 3l suggests that carbon existed on the C10-ZnSn(OH)_6_ surface. Additionally, the weight percentages of carbon in C5-ZnSn(OH)_6_, C10-ZnSn(OH)_6_, and C15-ZnSn(OH)_6_ samples were tested and found to be 5.99, 10.09, and 14.18, respectively, using an energy dispersive spectrometer, which is basically consistent with the theoretical results.

**Figure 4:**
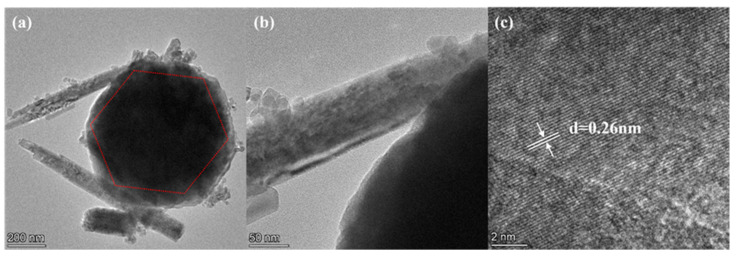
(**a**,**b**) TEM images and (**c**) HRTEM images of C10-ZnSn(OH)_6_ samples.

Figure 4 displays the TEM and HRTEM images of the C10-ZnSn(OH)_6_ samples, further confirming their octahedral morphology. The average side length of the octahedron is about 0.36 μm (Figure 4a). Additionally, one lattice plane of 0.26 nm is observed in HRTEM images, corresponding to (221) planes of the C10-ZnSn(OH)_6_, indicating an excellent crystallinity of the octahedron (Figure 4b,c). This analysis is consistent with the SEM and XRD results, further confirming the successful preparation of ZnSn(OH)_6_.

**Figure 5:**
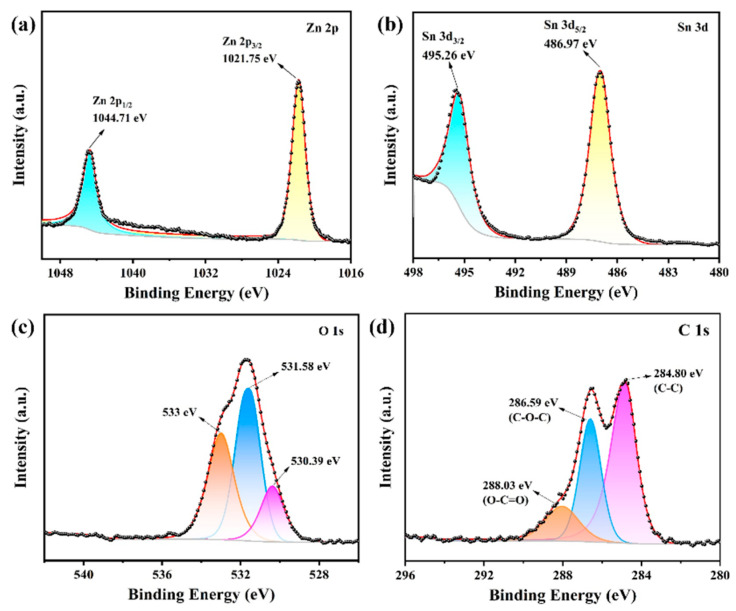
The XPS spectra of C10-ZnSn(OH)_6_ samples: (**a**) Zn 2p, (**b**) Sn 3d, (**c**) O 1s, and (**d**) C 1s.

The XPS analysis of the C10-ZnSn(OH)_6_ sample is shown in Figure 5. The whole spectra illustrate that Zn, Sn, O, and C exist in this sample. In the Zn 2p spectrum, the peaks at 1044.71 eV and 1021.75 eV binding energies correspond to 2p_1/2_ and 3p_3/2_ for Zn^2+^, respectively (Figure 5a) [26]. Similarly, in the Sn 3d spectrum, the binding energy peaks at 495.26 eV and 486.97 eV originated from 3d_3/2_ and 3d_5/2_ for Sn^4+^, respectively (Figure 5b) [27]. Additionally, three peaks are obtained by fitting the O1s peaks, where the peaks at 530.39 eV, 531.58 eV, and 533 eV correspond to lattice oxygen, defective oxygen, and surface adsorbed oxygen in C10-ZnSn(OH)_6_, respectively (Figure 5c) [28,29,30]. In the spectrum of C 1s, the characteristic peaks at 284.80 eV, 286.59 eV, and 288.03 eV are from C-C, C-O-C, and O-C=O bonds, respectively (Figure 5d) [31]. The XPS results further demonstrate the presence of carbon in C10-ZnSn(OH)_6_.

**Figure 6:**
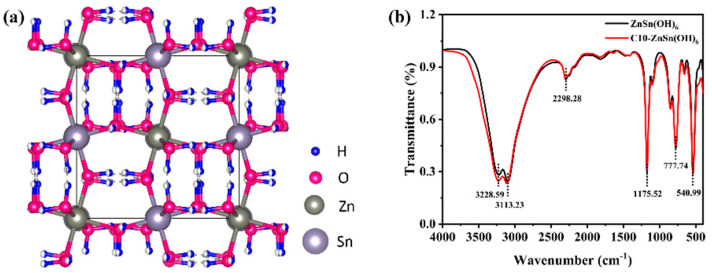
(**a**) The crystal structure of ZnSn(OH)_6_ sample and (**b**)FT-IR spectrum of C10-ZnSn(OH)_6_ and pure ZnSn(OH)_6_ samples.

The ZnSn(OH)_6_ crystal structure model is established through the crystallographic dataof its PDF card. The crystal structure of ZnSn(OH)_6_ illustrates that its surface contains abundant hydrophilic hydroxyl groups (Figure 6a). These hydroxyl groups would contribute to form H bonds with water molecules in humidity detection, facilitate ion mobility, and manifest macroscopically as a decrease in the resistance of sensors. Therefore, the content of hydroxyl groups in humidity-sensing materials is closely related to its sensitivity.

The microstructure and surface defects of materials play an essential role in sensing performance. Therefore, the samples were analyzed by FT-IR spectroscopy to determine further the functional groups on the surface, as shown in Figure 6b. The 3228.59 cm^−1^ and 3113.23 cm^−1^ spectra exhibit two broad peaks related to OH stretching vibration and H-bonded OH stretch, respectively [32]. The absorption peak near 2298.28 cm^−1^ may result from asymmetric stretching vibrations of atmospheric CO_2_ or CO_2_ adsorbed on the sample surface [33]. A strong absorption peak of Sn-OH bending vibrations is also observed at 1175.52 cm^−1^ [34]. The absorption peaks at 777.74 cm^−1^ and 540.99 cm^−1^ are attributed to the hydrogen bonding between water molecules and the Sn-OH stretching [35]. The FT-IR spectrum of the C10-ZnSn(OH)_6_ and the pure sample are basically consistent. The hydroxyl group is hydrophilic with a high affinity for water molecules. It provides mobile protons in different humidity environments, which increases electrical properties and ultimately affects the proton transport mechanism.

**Figure 7:**
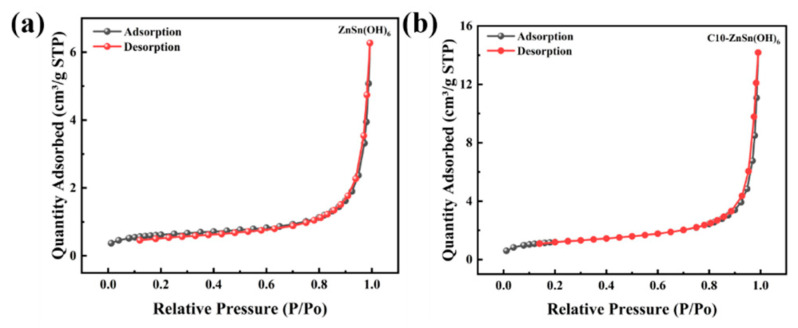
BET nitrogen adsorption–desorption curves of (**a**) ZnSn(OH)_6_ and (**b**) C10-ZnSn(OH)_6_ samples.

Nitrogen adsorption and desorption tests are conducted to further investigate the influence of carbon modification on ZnSn(OH)_6_ (Figure 7). The specific surface areas of pure ZnSn(OH)_6_ and C10-ZnSn(OH)_6_ are 2.14 m^2^/g and 4.24 m^2^/g, respectively. The C10-ZnSn(OH)_6_ exhibits a larger specific surface area. The higher specific surface area may provide more active sites on the material surface, which are beneficial to reactions and promote further response. Thus, it is inferred that the humidity-sensitive response of C10-ZnSn(OH)_6_ may be enhanced.

### 3.2. Humidity-Sensitive Performance

**Figure 8:**
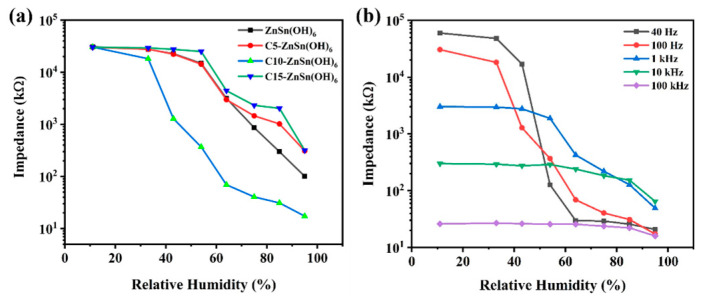
(**a**) The dependence of impedance on RH for pure and carbon/ZnSn(OH)_6_ sensors at 100 Hz and AC 1 V. (**b**) The dependence of impedance on RH for the C10-ZnSn(OH)_6_ sensor at various frequencies.

Figure 8a shows the impedance variation curves with ambient humidity for pure phase ZnSn(OH)_6_ and carbon/ZnSn(OH)_6_ sensors at 100 Hz and 1 V. The impedance response of the pure ZnSn(OH)_6_ sensor is two orders of magnitude in the humidity range of 11–95% RH. The impedance of the C10-ZnSn(OH)_6_ sensor decreases from 30,910 kΩ to 17.3 kΩ, realizing three orders of magnitude. The sensor response (S_R_) can be defined as *S_R_* = (*Ra* − *Rg*)/*Rg* × 100, where *Ra* represents the impedance at 11% RH and *Rg* represents the impedance at 95% RH [36]. Therefore, the response of ZnSn(OH)_6_, C5-ZnSn(OH)_6_, C10-ZnSn(OH)_6_, and C15-ZnSn(OH)_6_ sensors are calculated to be 30,595%, 9594%, 176,027%, and 9396%, respectively. Meanwhile, the C10-ZnSn(OH)_6_ sensor behaves with a good linearity. C5-ZnSn(OH)_6_ and C15-ZnSn(OH)_6_ sensors exhibit a relatively low response and poor linearity. Therefore, C10-ZnSn(OH)_6_ was an optimal choice for subsequent testing.

Figure 8b shows the relationship between impedance and relative humidity for the C10-ZnSn(OH)_6_ sensor at different frequencies (40 Hz, 100 Hz, 1 kHz, 10 kHz, and 100 kHz). The operating frequency significantly affects the humidity-sensitive performance. As the operating frequency increases, the humidity-sensitive response tends to level off, indicating that the polarization process in the water is smaller than the electric field change [37]. Overall, the humidity sensor exhibits an unstable humidity response at 40 Hz and a poor response at higher frequencies. Therefore, 100 H_Z_ was selected as the optimum operating frequency for subsequent measurement.

**Figure 9:**
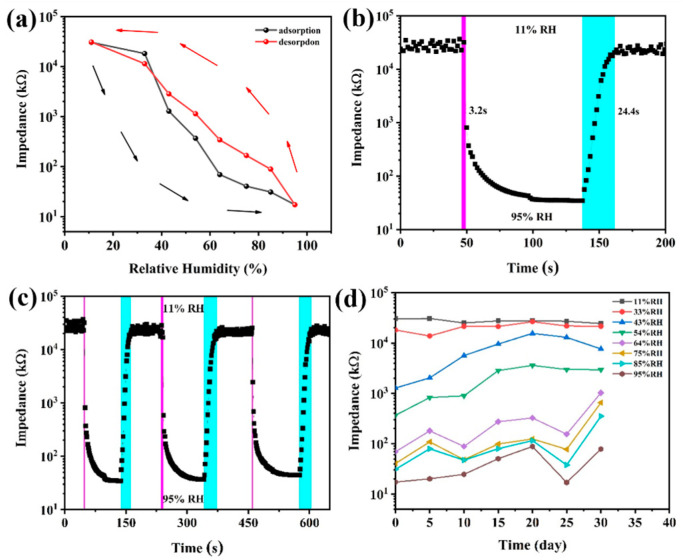
(**a**) Humidity hysteresis curve, (**b**) response/recovery time curve, (**c**) periodic response curve after consecutive cycles, and (**d**) long-term stability for the C10-ZnSn(OH)_6_ sensor.

Figure 9a presents a humidity hysteresis graph of the C10-ZnSn(OH)_6_ sensor. The sensor was placed in 11%, 33%, 43%, 54%, 64%, 75%, 85%, and 95% RH humidity ambient, and the impedance was recorded. Then, the sensor was placed in high to low humidity environments, in order, to obtain the hysteresis properties of the sensor. The calculation method for hysteresis is as follows: *γH* = ±Δ*H_max_*/2*F_FS_*, where Δ*H_max_* represents the maximum difference in impedance at the same relative humidity, and *F_FS_* represents the full scale output [38]. The impedance is recorded when stable, showing a hysteresis of about 13.5%. Hydrophilic hydroxyl groups can form hydrogen bonds with water molecules. During the adsorption process, the formation of hydrogen bonds helps to enhance the conductivity of the material. During the desorption process, as water molecules dissociate, hydrogen bonds gradually disappear, leading to a decrease in the material’s conductivity and an increase in impedance. This leads to the inconsistent response speed of the C10-ZHS humidity sensor during adsorption and desorption processes when relative humidity environment changes, resulting in the moisture hysteresis phenomenon.

The response/recovery time of the sensor is shown in Figure 9b. The response time is 90% of the total impedance change time of the sensor from 11% RH to 95% RH (as the red color shows). Similarly, the recovery time is required to reach a 90% change in total impedance during the desorption process from 95% RH to 11% RH (as the blue color shows). The results reveal that the C10-ZnSn(OH)_6_ sensor’s response time is 3.2 s, and the recovery time is 24.4 s. To explore the repeatability of the sensor, the response and recovery characteristics are tested after consecutive cycles (Figure 9c). The C10-ZnSn(OH)_6_ sensor shows high stability and repeatability over several successive cycles. The average response time of the device is 4.2 s, and the recovery time is 27.8 s. Long-term stability is a crucial humidity-sensitive characteristic that reflects sensors’ practical application performance. To investigate the sensors’ stability, the impedance fluctuation with the humidity of the C10-ZnSn(OH)_6_ sensor was explored for 30 days, with measurement every 5 days at intervals from 11% to 95% RH (Figure 9d). It was found that the C10-ZnSn(OH)_6_ sensor was stable at low relative humidity, while there was some fluctuation at high relative humidity, showing relatively reliable stability overall.

In Table 1, the response/recovery time of the C10-ZnSn(OH)_6_ humidity sensor fabricated in this work was compared with recent research. It can be seen that the C10-ZnSn(OH)_6_ humidity sensor exhibits excellent response/recovery characteristics.

### 3.3. Mechanistic Analysis of the Sensors

**Figure 10:**
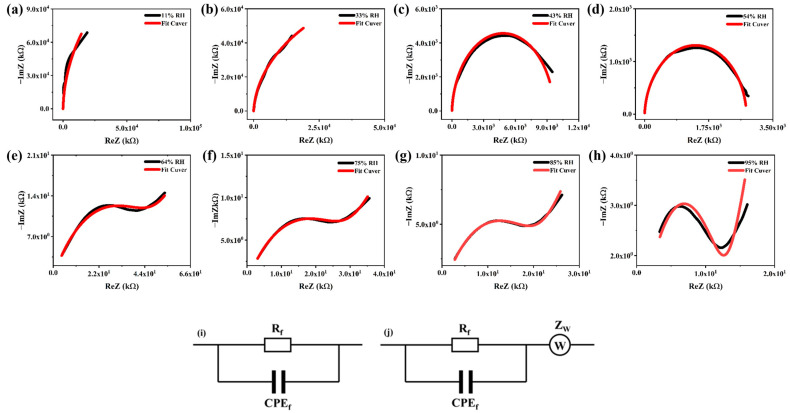
(**a**–**h**) CIS and equivalent circuit fitting curves of the C10-ZnSn(OH)_6_ humidity sensor under different relative humidity environments and (**i**,**j**) the corresponding equivalent circuits.

To further investigate the humidity sensing mechanism, the complex impedance spectrum (CIS) of the sensor was obtained at 11%, 33%, 43%, 54%, 64%, 75%, 85%, and 95% RH, and equivalent circuits were fitted to analyze its sensing characteristics. The C10-ZnSn(OH)_6_ sensor impedance variation was measured at different humidity ambient from 11% RH to 95% RH over the operating frequency range of 40 Hz to 100 kHz (Figure 10). Figure 10a,b shows that at a relatively low humidity of 11% RH and 33% RH, the CIS curve is an arc with a large curvature. Under low humidity conditions, water molecules in the environment first exist on the sample surface, and then are ionized under a electrostatic force (H_2_O = H^+^ + OH^−^). As the number of hydrophilic hydroxyl increases, a hydroxyl group is formed. When hydroxyl groups contact water molecules, the negative charge of the oxygen in the hydroxyl group interacts with the positive charge of the hydrogen in the water molecule, forming hydrogen bonds. The formation of hydrogen bonds enhances the interaction force between hydroxyl groups and water molecules, thereby promoting their binding with water molecules and enhancing the adsorption of sensitive materials towards water molecules [42]. As shown in Figure 10c,d, the adsorbed water molecules gradually increase with the increase in humidity (43–54%RH). The proton (H^+^) combines with the water molecules adsorbed on it through the hydroxyl group to form H_3_O^+^ [9], prone to leakage conductive flow, thus giving the CIS curve as a semicircular shape. The primary carrier in this stage is H_3_O^+^. Figure 10e–h depicts that as the relative humidity further increases (64%, 75%, 85%, and 95% RH), the CIS curve consists of a semicircle at high frequencies and a trailing line at low frequencies. At this point, the accumulation of water molecules formed a physical adsorption layer, and a liquid water layer grew up on the surface of the sensing sample [43]. On the liquid water layer, the H_3_O^+^ releases a proton to an adjacent water molecule, releasing a proton to its following water molecule, realizing the free movement of protons in the liquid water layer. This is the Grotthuss chain reaction (H_2_O + H_3_O^+^→ H_3_O^+^ + H_2_O) [44], occurring at low frequencies. Therefore, a straight line is observed at the end of the CIS curve. Thus, in high-humidity environments, both H_3_O^+^ and protons act as the leading carriers for conduction.

The equivalent circuit diagram of CIS for C10-ZnSn(OH)_6_ is presented in Figure 10i,j. At 11–54% RH, the semi-circular curve of CIS represents a sensing mechanism by dielectric correlation theory [9]. An equivalent circuit is fitted as a resistor (R_f_) connected in parallel with a constant phase element (CPE_f_), as shown in Figure 10i. The equivalent circuit is composed of Rf and CPE_f_ in parallel and then in series with the Warburg impedance (Z_w_) under a high humidity environment (64–95% RH), as shown in Figure 10j. Therefore, the C10-ZnSn(OH)_6_ sensor shows different humidity sensing mechanisms at low and high relative humidity. In addition, according to Table 2, it can be concluded that the maximum fitting error of the equivalent circuit is less than 2.1%, demonstrating a good fitting effect.

**Figure 11:**
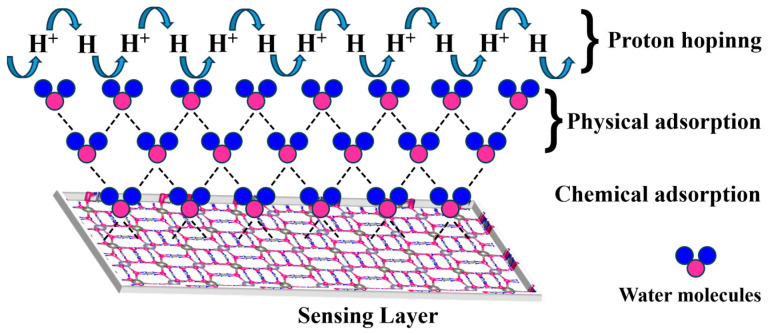
Sensing mechanism diagram of humidity sensors.

For the C10-ZnSn(OH)_6_ sample, the introduction of carbon mainly affects the surface state of ZnSn(OH)_6_; the surface of carbon modified samples appears rougher with a larger specific surface area, which is more conducive to the active sites on the surface to fully contact water molecules and enhance their adsorption capacity for water molecules. When the C10-ZnSn(OH)_6_ sensoris working, H^+^ and H_3_O^+^ are still the main carriers for conductivity. The sensing schematic diagram of the C10-ZnSn(OH)_6_ humidity sensor is shown in Figure 11. In low relative humidity environments, the sensing layer of C10-ZnSn(OH)_6_ mainly adsorbs water molecules through chemical adsorption. As the relative humidity increases, it gradually transforms into the physical adsorption of water molecules. In a high relative humidity environment, the Grotthuss chain reaction is formed, and protons skip to conduct.

## 4. Conclusions

The synthesis and characterization of pure ZnSn(OH)_6_ and carbon/ZnSn(OH)_6_ are discussed in this research. ZnSn(OH)_6_ humidity sensors modified with different carbon contents were fabricated, and their sensing performance was explored. By introducing carbon, the roughness of the surface of ZnSn(OH)_6_ grains was increased, resulting in a doubling of their specific surface area and an increase in active sites on the surface of C10-ZnSn(OH)_6_, which is beneficial for the adsorption of water molecules. The C10-ZnSn(OH)_6_ sensor exhibits a high impedance response close to three orders of magnitude, a humidity hysteresis of 13.5%, a fast response time (3.2 s), and a recovery time of 24.4 s. FT-IR and crystal structure analyses reveal that the C10-ZnSn(OH)_6_ sample surface is rich in hydroxyl groups, which contribute significantly to the adsorption of water molecules. The humidity sensor’s possible sensing mechanism was also analyzed using the AC complex impedance spectrum with a simulated equivalent circuit. In conjunction with ionic conductivity theory, protons and hydrated hydrogen ions act as the main conducting carriers, with proton ions playing an essential role in low- and high-humidity environments. These results demonstrate the superior performance of ZnSn(OH)_6_ for effective humidity detection and that carbon modification significantly improves the humidity performance of pure ZnSn(OH)_6_.

## Figures and Tables

**Table 1:** A comparison of the response/recovery time of humidity sensors across different studies.

Material	Measurement Range	T_response_	T_recovery_	Ref.
SnO_2_-TiO_2_	11–95% RH	19.1 s	181 s	[5]
c-IPN polymer	30–90% RH	38 s	70 s	[6]
Cu_2_ZnSnS_4_	11–97% RH	105 s	36 s	[39]
Sn-doped NiO	10–90% RH	43 s	38 s	[40]
PANIHCl/ZnO	10–90% RH	13 s	171 s	[41]
C10-ZnSn(OH)_6_	11–95% RH	3.2 s	24.4 s	This work

**Table 2:** Errors in CIS curve equivalent circuit fitting for the C10-ZnSn(OH)_6_ sensor.

Relative Humidity	11% RH	33% RH	43% RH	54% RH	64% RH	75% RH	85% RH	95% RH
Overall error rate	1.17%	1.29%	2.09%	1.83%	0.91%	0.38%	0.25%	0.87%

## Data Availability

Data is available upon request.
